# Canine D_163_-PrP polymorphic variant does not provide complete protection against prion infection in small ruminant PrP context

**DOI:** 10.1038/s41598-021-93594-x

**Published:** 2021-07-12

**Authors:** Alba Marín-Moreno, Juan Carlos Espinosa, Patricia Aguilar-Calvo, Natalia Fernández-Borges, José Luis Pitarch, Lorenzo González, Juan María Torres

**Affiliations:** 1 Centro de Investigación en Sanidad Animal, CISA-INIA, Valdeolmos, Madrid Spain; 2 Animal Health and Veterinary Laboratories Agency (AHVLA), Penicuik, Midlothian UK

**Keywords:** Prions, Neurological disorders, Neurodegeneration, Prion diseases

## Abstract

E/D_163_ polymorphism of dog prion protein (PrP) has been recently proposed as the variant responsible for canid prion resistance. To further investigate the protective role of this variant against prion replication, the transgenic mouse model OvPrP-Tg532 expressing sheep/goat PrP carrying the substitution D_162_ (equivalent to D_163_ position of dog PrP) was generated and intracranially inoculated with a broad collection of small ruminant prion strains. OvPrP-Tg532 mice showed resistance to classical bovine spongiform encephalopathy (BSE) from sheep and some classical scrapie isolates from sheep and goat but were susceptible to ovine atypical L-BSE and numerous classical scrapie isolates. Strikingly, some of these classical scrapie isolates showed a shift in their prion strain properties. These results suggest that other PrP residues apart from E/D_163_ variant of dog PrP or factors distinct than PrP may participate in prion resistance of canids and that different factors may be required for D_162_ sheep PrP to provide effective protection to sheep against ruminant prions.

## Introduction

Prion diseases or transmissible spongiform encephalopathies (TSEs) are fatal neurodegenerative diseases. The key molecular event in the pathogenesis of prion diseases is the post-translational modification of the host encoded cellular prion protein PrP^C^ into a disease-associated isoform called PrP^Sc^^1^. PrP^Sc^ self-catalyzes its formation by recruiting and transforming PrP^C^ into PrP^Sc^ aggregating and acting as a template to produce newer PrP^Sc^ particles^2^. PrP^Sc^ suffers a change in its secondary structure, richer in β-sheet content. Such transformation modifies the protein biochemical features. While PrP^C^ is monomeric, protease-sensitive and soluble in non-ionic detergents^3^, PrP^Sc^ has a high tendency to aggregate, is partially resistant to proteases and insoluble^1^.

TSEs affect a wide range of mammal species like humans and other species in the human food chain. Human prion diseases include genetic, infectious and sporadic disorders, like Creutzfeldt-Jakob disease (CJD), kuru, Gerstmann- Straussler-Scheinker syndrome (GSS), fatal familial insomnia (FFI) and variably protease-sensitive prionopathy (VPSPr)^4^. Animal prion diseases encompass scrapie in sheep and goats, chronic wasting disease (CWD) in cervids, bovine spongiform encephalopathy (BSE), transmissible mink encephalopathy (TME)^5^ and camel prion disease (CPD)^6^.

Experiments in PrP transgenic mice proved that the efficacy of PrP^C^ conversion into PrP^Sc^ mainly rely on the homology grade among the primary sequence of PrP^Sc^ and PrP^C^^7^, a concept known as “species barrier”. A PrP^C^-PrP^Sc^ interaction sharing the primary sequence is homologous and the efficiency of conversion will be fairly high. By contrast, amino acid differences promote a heterologous interaction which is associated with a decreased efficiency of PrP^C^ transformation^8^.

PrP^C^ amino acid sequence has been well conserved through evolution^9^, pointing to an important role of PrP^C^ although its primary function remains unknown. Sequence alignments of mammalian PrPs along with reports of susceptibility/resistance of certain animals to TSEs have resulted in the identification of several amino acids with key roles in susceptibility/resistance to prions. Of special importance is the residue 163 of dog PrP, which can harbor aspartate (D) or glutamate (E), instead of the asparagine (N) encoded in other mammals. This variant has been recently attributed to being the main, if not the only, responsible for the prion resistance of canids^10,11^. Transgenic mice expressing dog PrP as well as mouse PrP harboring the equivalent D_158_ variant were resistant to prion infection with three mouse-adapted prion strains^10,11^. Coexpression of both wild-type and mutated D_158_ PrP variant in transgenic mice inoculated with mouse-adapted prions increases survival times indicating that this substitution confers a dominant-negative effect on PrP^12^. Finally, transgenic mice expressing N_163_-dog PrP were susceptible to sheep classical BSE prions in vivo and to sheep and cattle classical BSE prions in vitro^11^.

It is well known that prion strains also affect prion conversion. Prion strains are defined as conformational variants which show distinct prion-disease phenotypes when transmitted to hosts with identical PrP sequence^13^. So, the efficiency of prion replication is not only controlled by the identity between the PrPs involved in the process but also by the prion strain, which renders this replication favorable only when the PrP^Sc^ structure is one of the possible that host PrP^C^ can adopt as stated by the conformational selection model^14^. Thus, resistance/susceptibility to TSEs will always be dependent on the prion strain that is being studied in each case.

This study further investigates the protective role exerted by the dog PrP D/E_163_ polymorphic variant in the sheep/goat PrP context towards prion replication. For that purpose, a novel transgenic mouse model called OvPrP-Tg532 was generated. These transgenic mice expressed small ruminant PrP carrying the substitution D_162_ (equivalent to D/E_163_ position of dog PrP) and were intracranially inoculated with sheep/goat prion isolates representing different scrapie and BSE strains. The use of isolates from the same species (sheep/goat) avoided the species barrier facilitating prion transmission to precisely test the effect of the D_162_ substitution. Animals were only resistant to some isolates, suggesting that although the dog PrP D/E_163_ variant plays an important role in canid resistance to prion diseases, distinct PrP residues or factors other than PrP must collaborate in this phenomenon.

## Results

### Generation of OvPrP-Tg532 transgenic mice

**Figure 1:**
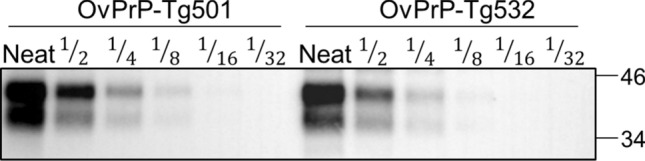
Brain PrP^C^ expression in OvPrP-Tg501 and OvPrP-Tg532 mice. Immunoblotting of PrP^C^ detected using 12B2 mAb. Neat sample (10% brain homogenates) and serial dilutions were loaded on 12% Bis–Tris gels. The figure illustrates a representative set of three independent experiments. Relative molecular mass in kDa at the left side. Full-length blots/gels are presented in Supplementary Figure 1.

The same plasmid vector used to generate OvPrP-Tg501 mouse line^15^ was used to generate the new transgenic mouse line as described in the Materials and Methods section. Several founder lines hemizygous for the transgene (D_162_GoPrP^+/-^) were obtained. Founder animals (muPrP^+/-^ D_162_GoPrP^+/-^) were crossed with *prnp* null mice (muPrP^-/-^) to obtain transgenic hemizygous lines in a murine *prnp* null background (muPrP^-/-^ D_162_GoPrP^+/-^). The absence of the murine *prnp* was determined by PCR using specific primers. Then, hemizygous mice were crossed to produce homozygous animals (muPrP^-/-^ D_162_GoPrP^+/+^). At this point, PrP^C^ expression level was determined in brain homogenates of mice by serial dilution in western blotting (WB) and compared to OvPrP-Tg501 brain PrP^C^ expression levels. OvPrP-Tg532 mouse line was selected based on the fact that they showed brain PrP^C^ levels and an electrophoretic profile similar to that of OvPrP-Tg501 mice (Fig. 1). OvPrP-Tg532 mice reached the end of their lifespan (> 650 days post-inoculation) with neither evidence of spontaneous prion disease nor behavioral defects.

### Susceptibility of OvPrP-Tg532 transgenic mice to several prion strains

**Figure 2:**
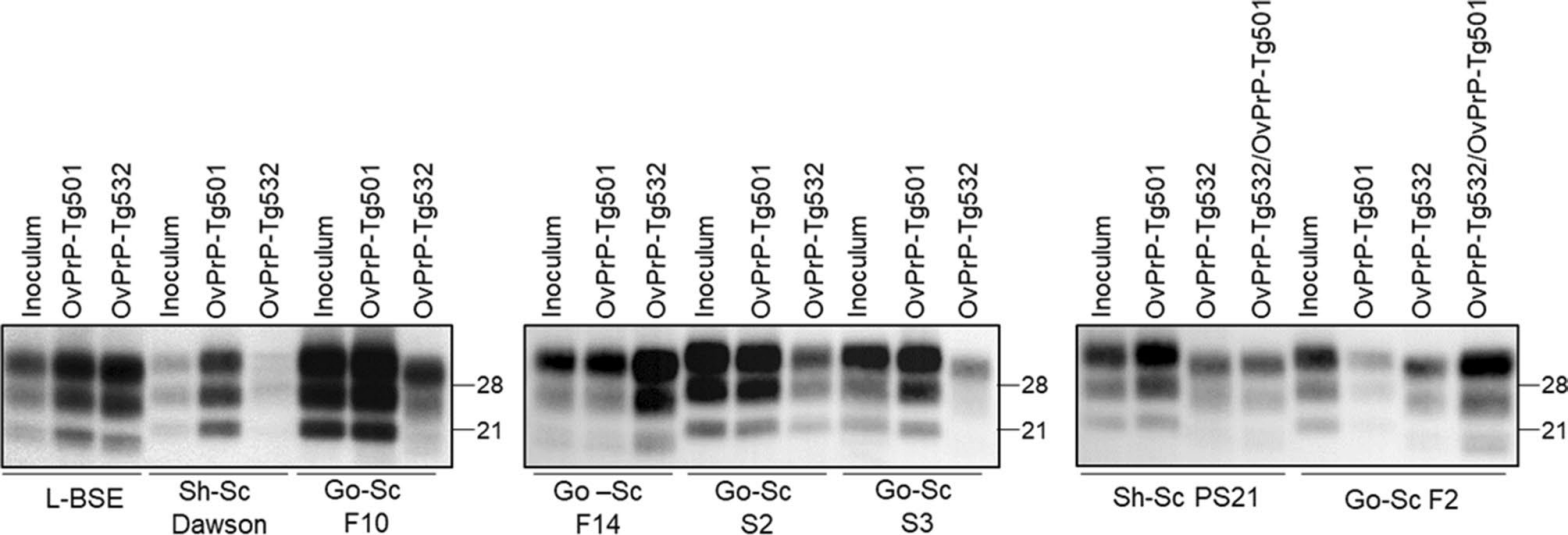
Comparison between inocula and brain PrP^res^ in OvPrP-Tg501 and OvPrP-Tg532 mice. Immunoblotting of PrP^res^ detected using Sha31 mAb. The figure illustrates a representative set of three independent experiments. Relative molecular mass in kDa at the left side. Full-length blots/gels are presented in Supplementary Figure 1.

OvPrP-Tg532 mice were intracranially challenged with isolates of classical and atypical BSE adapted to the ovine PrP sequence (Table 1). Classical BSE from experimentally infected goat and sheep, which is well known to successfully propagate into OvPrP-Tg501 mice expressing the wild type small ruminant PrP^15^, did not infect OvPrP-Tg532 mice expressing the mutated D_162_-PrP (Table 1). Analysis of brain proteinase K resistant PrP (PrP^res^) by WB as well as the histological examination of brain tissue were negative (data not shown). By contrast, OvPrP-Tg532 mice got infected with ovine adapted L-BSE isolate (Table 1) showing the same brain PrP^res^ biochemical profile by WB (Fig. 2A) and the same histological features (data not shown) as OvPrP-Tg501 animals challenged with the same inocula. OvPrP-Tg532 mice were also inoculated with several scrapie isolates from sheep and goat. These isolates were collected in different European countries representing the variety of classical scrapie strains circulating in Europe. OvPrP-Tg532 mice proved to be susceptible to all scrapie isolates except from the Italian isolates 198 and I9 from sheep and goat respectively as well as to classical BSE prions as previously mentioned (Table 1). Detection of PrP^res^ by WB and histological analysis of the brains of these latter mice produced negative results (data not shown). In addition, sheep Dawson and goat S2 isolates did not produce 100% attack rates (Table 1).

**Table 1:** Prion transmission in OvPrP-Tg532 and OvPrP-Tg501 mice.

Prion isolate	Origin (Supplier^a^)	Goat PrP genotype^b^	Description	Mean survival time ± SD^c^ (n/n_0_^d^)	
				OvPrP-Tg532 (D_162_)	OvPrP-Tg501 (N_162_)
Go-BSE^15,37^	France (INRA)	wt	Natural case of cattle BSE inoculated in goat	> 650 (0/5)^e^	346 ± 16 (6/6)
Sh-BSE^31^	France (INRA)	wt	Natural case of cattle BSE inoculated in sheep	> 650 (0/5)^e^	485 ± 62 (7/7)
L-BSE^32^	Poland (CISA)	wt	Natural case of atypical L-BSE passaged into OvPrP-Tg501	> 405 (5/5)^f^	289 ± 46 (5/5)
Sh-Sc PS21^33^	France (ENVT)	wt	Natural case	270 (6/6)	222 ± 5 (6/6)
Sh-Sc PS21/OvPrP-Tg532	Spain (CISA)	D_162_	Natural case of classical scrapie passaged into OvPrP-Tg532	-	186 ± 8 (7/7)
Sh-Sc Dawson^34^	France (CISA)	wt	Natural case of classical scrapie passaged into OvPrP-Tg501	452 (1/5)	259 ± 13 (6/6)
Sh-Sc 198–9^35^	Italy (ISS)	wt	Natural case	> 650 (0/5)^e^	537 ± 13 (7/7)
Go-Sc I9^36,37^	Italy (IZSTO)	R/H_154_; S/P_240_	Natural case	> 650 (0/5)^e^	600 ± 43 (5/5)
Go-Sc F10^15^	France (INRA)	S/P_240_	Natural case	301 (4/4)^f^	449 ± 19 (6/6)^g^
Go-Sc F14^36,37^	France (CISA)	S/P_240_	Natural case of classical scrapie passaged into OvPrP-Tg501	301 ± 25 (5/5)	287 ± 94 (5/5)
Go-Sc F2^15^	France (INRA)	S/P_240_	Natural case	> 650 (6/6)^e^	239 ± 21 (4/4)^g^
Go-Sc F2/OvPrP-Tg532	Spain (CISA)	D_162_	Natural case of classical scrapie passaged into OvPrP-Tg532	–	208 ± 15 (6/6)
Go-Sc S2^15^	Spain (CISA)	wt	Natural case of classical scrapie passaged into OvPrP-Tg501	441 ± 77 (4/5)	228 ± 15 (6/6)^g^
Go-Sc S3^15^	Spain (CISA)	wt	Natural case of classical scrapie passaged into OvPrP-Tg501	233 ± 15 (5/5)	221 ± 16 (6/6)^g^
Healthy goat brain^15^	France (INRA)	wt	Brain from an uninfected goat	> 650 (0/6)^e^	> 650 (0/6)^e^

^a^CISA, Centro de Investigación en Sanidad Animal, Madrid, Spain; ENVT, École Nationale de Veterinaire, Toulouse, France; INRA, French National Institute for Agricultural Research, Nouzilly, France; ISS, Istituto Superiore di Sanitá, Rome, Italy; IZSTO, Istituto Zooprofilattico Sperimentale del Piemonte, Italy; Roslin, The Roslin Institute and Royal (Dick) School of Veterinary Studies, University of Edinburgh, United Kingdom.

^b^The wild-type (wt) goat prion protein genotype is A_136_R_154_P_240_/ A_136_R_154_P_240_.

^c^Mean survival time ± standard deviation in dpi.

^d^Attack rate expressed as the relation of brain PrP^res^ positive animals (n) and the total number of inoculated animals (n_0_).

^e^Animals were sacrificed because the experimental endpoint was reached without showing clinical signs of prion disease.

^f^Dead due to causes not related to prion disease.

^g^Previously published in reference number 37.

Nearly all successfully transmitted isolates conserved the original PrP^res^ electrophoretic profile in WB (Fig. 2) and the histological characteristics were indistinguishable from the ones displayed in control OvPrP-Tg501 animals (data not shown). However, scrapie isolates PS21 from sheep and F10, F14, F2 and S3 from goat displayed a change in their brain PrP^res^ signature in WB which shifted the 21 kDa of the non-glycosylated band to a 19 kDa molecular weight (Fig. 2). Sheep scrapie PS21 and goat scrapie F2 were selected for further study and second passage were performed in OvPrP-Tg501 (Table 1). Brain PrP^res^ from these animals retained the 19 kDa molecular weight of the non-glycosylated band (Fig. 2).

**Figure 3:**
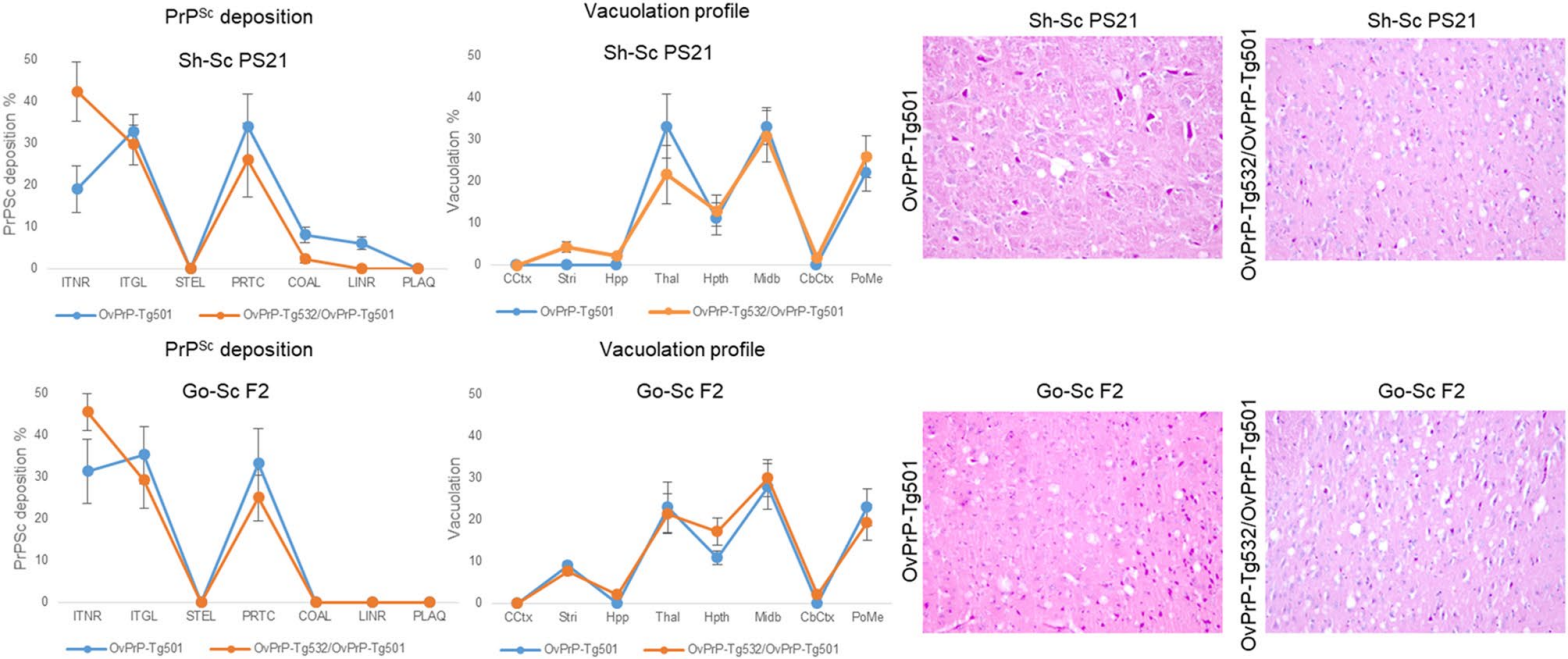
Histological analysis of the brains of mice inoculated with Sh-Sc 21 and Go-Sc F2 inocula. Brain lesion profiles were performed using hematoxylin and eosin staining using samples from at least 4 inoculated mice. The brain areas analyzed were cerebral cortex (CCtx); striatum (Stri); hippocampus (Hpp); thalamus (Thal); hypothalamus (Hpth); Midbrain (Midb); Cerebelar cortex (CbCtx) and pons/medulla oblongata (PoMe). The representative pictures showing vacuolization were taken in the midbrain section. PrP^Sc^ deposit profile was performed by IHC with 2A11 mAb. The magnitude of PrP^Sc^ accumulation was scored using samples from at least 4 inoculated mice in the mentioned above brain areas considering the following PrP^Sc^ types: intraneuronal (ITNR), intraglial (ITGL, intra-microglial and intra-astrocytic combined), stellate (STEL), fine particulate (PRTC), coalescing (COAL), linear (LINR) and plaques (PLAQ, vascular and non-vascular combined).

Histologically, once transmitted into the OvPrP-Tg532 and back into OvPrP-Tg501 mice the lesion profiles for sheep scrapie PS21 and goat scrapie F2 were slightly modified (Fig. 3). The PrP^Sc^ deposition profile showed an increase in the intraneural deposition for both OvPrP-Tg532 adapted inocula (sheep scrapie PS21 and goat scrapie F2) transmitted back into OvPrP-Tg501 while the severity of the other PrP deposits decreased (Fig. 3).

## Discussion

The dog-PrP D/E_163_ polymorphic variant has been recently proposed as responsible for the prion resistance of canids^10,11^. Such variant was found to protect against mouse-adapted strains RML, 22L and 301C in transgenic mice expressing the mouse D_158_ PrP variant equivalent to the dog D_163_^10^. Coexpression of both the wild-type and mutated PrPs in transgenic mice inoculated with mouse-adapted prion strains increased mice survival time^12^. Later, transgenic mice expressing dog PrP harboring the E_163_ polymorphic variant were resistant to classical and atypical L-BSE and classical and atypical scrapie prions^11^. Moreover, once the N_163_ mutation was introduced in transgenic mice expressing dog PrP, animals were susceptible to sheep classical BSE in vivo and to cattle and sheep classical BSE prions in vitro^11^. To further study the role exerted for this polymorphic variant in the susceptibility/resistance of prions, we generated the OvPrP-Tg532 mouse line. This line expresses physiological levels of small ruminant PrP harboring the mutation D_162_, analogue to the D/E_163_ polymorphic variant of dog PrP. These mice were intracranially inoculated with a broad panel of prion isolates with different strain features.

OvPrP-Tg532 proved to be resistant to classical BSE from ovine and caprine origin (Table 1) while its control counterpart model (OvPrP-Tg501) was susceptible with 100% attack rates. This result is in line with the previously published mouse model expressing the D_158_-mouse PrP, in which the BSE mouse-adapted strain 301C was unable to cause disease after intracerebral challenge^10^. However, dog PrP, which expresses in a natural manner the “resistant” D_163_ could eventually be misfolded in vitro by BSE and BSE-derived prions that retained their ability to infect bovine PrP transgenic mice^16^. Altogether, suggests the molecular compatibility between D_163_ dog PrP variant and classical BSE PrP^Sc^ at least in vitro. An in silico analysis of this acidic substitution revealed an electrostatic potential change on the surface of PrP when compared to its counterpart amino acid (asparagine). The acidic properties conferred by D/E residue disturb this region introducing a positively charged residue that might block the PrP conversion^10^.

However, when searching for “universal” PrP sequences that could drive resistance against prions it is important to bear in mind that susceptibility to TSEs depends on the *PRNP* genotype and the infectious strain^17,18^. Thus, a wide panel of prion isolates was inoculated in the above mentioned small ruminant transgenic mice to test the role of the singular D_162_ mutation. The two Italian scrapie isolates included in this study, which showed long incubation times in OvPrP-Tg501 (Table 1)^15^, were not able to infect D_162_ OvPrP-Tg532 mice. Two possible explanations could account for this fact. As these strains are classified as slow replicating prions, the PrP containing the D_162_ mutation may just elongate the survival times making the disease not able to appear within the mouse lifespan. A not fully protective effect but a significant delay in disease manifestation was also found in the highly prion susceptible bank vole PrP transgenic mouse model in which this substitution was introduced^12^. In this case, the substitution could trigger a protective effect by inducing protein alterations which attenuate the rate of fibril formation and the stability of newly formed fibrils^10,19,20^. Alternatively, a complete resistance against these Italian scrapie isolates could explain these results. In fact, in the mouse context, the D_158_ mouse model was also completely resistant to the mouse scrapie adapted strains RML and 22L^10^. With the aim of reducing incubation times of these slow replicating scrapie strains in the OvPrP-Tg532 model (that could interfere with a polymorphic barrier induced by the substitution D_162_), second passages will be necessary.

L-BSE and the non-Italian scrapie isolates tested in this panel were able to produce disease in the OvPrP-Tg532 mouse model with attack rates close to 100%. However, slightly longer incubation periods were found in almost all the cases when compared to OvPrP-Tg501 control model (Table 1). These results highlight the importance of using a wide diversity of prion isolates to test the role of a singular PrP mutation in the prion susceptibility/resistance. Therefore, the mutation D_162_ does not provide total resistance against prion infection in the small ruminant PrP context; it depends on the prion strain tested on each case. Although the successful first passage already shows that the D_162_ variant in sheep PrP does not provide complete resistance to these prion strains, the second passages would be very informative. Once fully adapted to replication in both D_162_ OvPrP-Tg532 and N_162_ OvPrP-Tg501 models, a proper comparison of the transmission properties among the two models could be done.

In addition, for sheep scrapie PS21 and goat scrapies F2, F10, S2 and S3 isolates, the molecular weight of the non-glycosylated PrP band changed from 21 to 19 kDa (Fig. 2). This shift in the strain characteristics was permanent even after transmission back into the OvPrP-Tg501 mice and solely caused by the D_162_ mutation.

**Figure 4:**
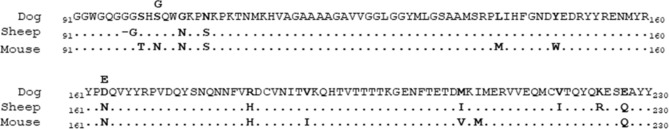
Dog, mouse and sheep PrP sequence alignment. PrP amino acid alignment from residue 91 to 230 of dog PrP from dog, sheep and mouse species. The dog amino acids that are different in the other two species are indicated in bold. Accession numbers: dog PrP (FJ870767.1); ovine PrP (NP001009481.1) mouse PrP (NP035300).

There is no report of TSE infection in canids and the D/E_163_ polymorphic variant was pointed as responsible for this even in the mouse PrP context^10,11^. However, we prove that the phenomenon of susceptibility/resistance to prions is strongly strain-dependent and the reports in the mouse PrP context^10,11^ included other prion strains. In addition, the transmission barrier in the dog brain can be overcome using the PMCA technique for classical BSE prion agent^16^. These experiments were done using dog brain expressing the D_163_ PrP polymorphic variant, the same we introduced in the sheep PrP sequence. However, the transgenic mouse line that was found to be resistant to prion infection using a variety of strains, including cattle and sheep classical BSE, expressed the E_163_ PrP polymorphic variant^11^. Within the *Canidae* family, the D_163_ PrP variant is present in several species while the E_163_ PrP is restricted to domestic dogs. Thus suggesting that the D_163_ variant could pose some resistance to prion infection in a strain dependent manner, while the E_163_ variant may be a more robust protector. Although canids are resistant to TSEs in natural conditions, its PrP can be misfolded in vitro by the classical BSE agent^16^ whilst the present study points that its resistance cannot be fully attributed to this polymorphic allele since the introduction of the equivalent D_162_ mutation in sheep PrP did not confer absolute prion resistance. However, it must be taken into account that D_162_-sheep PrP is not analogue to the dog PrP sequence and therefore other factors may be required for D_162_-sheep PrP to provide effective protection to sheep against ruminant prions. In addition, other canid PrP residues, or even other genetic factors different than PrP may be involved in fully canid prion resistance. A multiple protein sequence alignment done for mouse, sheep and dog PrP reveals that few amino acid residues apart from D/E163 polymorphism in dog PrP are distinct between these three species (Fig. 4). Since D_158_ mice were found resistant to the three prion strains tested^10,12^, amino acid differences between dog and mouse PrP did not account for the prion resistance, at least for the strains tested there. However in the case of our OvPrP-Tg532 mice, 7 amino acid positions differ with the dog PrP sequence and these positions may also be responsible for canid resistance and/or for sheep susceptibility to prion infection (Fig. 4). Little is known from other genetic factors other than PrP affecting prion disease progression. Quantitative trait locus (QTL) mapping, the crossing of heterogeneous stock mice, expression profiling and human genome wide association studies (GWAS) have pointed to the existence of non-PrP genetic modifiers (reviewed in 21). The role that these genes and their coding proteins may pose in prion propagation is not known but they generally belong to neurodegeneration, immune response and protein folding and degradation pathways^21^. It has also been reported that susceptibility to prion strains is mainly governed by host PrP^C^ while other host factors modulate strain features^22^.

With the reports existing up to now, the D_162_ mutation could have been considered interesting for the sheep and goat breeding selection programs conducted to reduce classical scrapie incidence within Europe. Although this polymorphism does not naturally exist in sheep and goats, genetically modified animals could have been produced. Unfortunately, our study demonstrates that D_162_ substitution does not provide total resistance against prion infection in the small ruminant PrP context, discarding this mutation as suitable for sheep and goat breeding selection programs.

## Methods

### Ethics statement

Animal experiments were carried out in accordance with the recommendations of the Code for Methods and Welfare Considerations in Behavioral Research with Animals (Directive 86/609EC and 2010/63/EU) and all efforts were made to minimize suffering. Experiments were approved by the Committee on the Ethics of Animal Experiments (CEEA) of the Spanish Instituto Nacional de Investigación y Tecnología Agraria y Alimentaria (INIA); Permit Number: CEEA2009/007. The reporting of the animal experiments in this manuscript follows the recommendations in the ARRIVE guidelines.

### OvPrP-Tg532 transgenic mice generation

OvPrP-Tg532 mouse line expressing D_162_ small ruminant PrP about onefold the PrP expression level in goat brain on a mouse PrP null background has been generated as previously described^15^.

The pMo-GoPrP.Xho plasmid used for the generation of OvPrP-Tg501 mice expressing the wild type small ruminant PrP^15^ was used as the template for directed mutagenesis reaction. pMo-GoPrP.Xho plasmid contains the goat PrP inserted in the MoPrP.Xho expression vector^23^. Goat and sheep PrP share the same primary sequence just differing in the nucleotide sequence for certain amino acids. The MoPrP.Xho expression vector contains the murine PrP promoter, exon 1, intron 1, exon 2, and 3’-untranslated sequences. The plasmid was mutated to generate a D_162_-PrP plasmid by using a QuikChange II XL kit (Stratagene, CA) with specific oligonucleotides (5 ‘-GTACCGTTACCCCGACCAAGTGTACTACAGACCAG-3 ‘ and 5’- CTGGTCTGTAGTACACTTGGTCGGGGTAACGGTAC-3 ‘), following the manufacturer’s instructions.

The transgene was excised from the expression vector by use of NotI. The corresponding gel slice was excised and digested using β-agarase I (New England Biolabs) as described by the manufacturer. Purified DNA was resuspended in TE (10 mM Tris, pH 7.4, 0.1 mM EDTA) at a final concentration of 2 to 6 ng/ml and microinjected into pronuclear-stage embryos collected from superovulated B6CBAF1 females mated with PrP null 129/Ola males^24^. DNA was extracted from founders’ tail biopsy by use of an Extract-N-Amp tissue PCR kit (Sigma-Aldrich) following the manufacturer’s instructions. The transgene presence in founders was identified by PCR amplification using specific primers 5’-CATTCTGCCTTCCTAGTGGTACC-3’ and 5’-GCTTGTTCCACTGACTGTGGC-3’. MoPrP^+/-^ D_162_GoPrP^+/-^ founders were backcrossed with homozygous PrP null animals (MoPrP^-/-^) to obtain mice hemizygous for the mutation which lack murine PrP expression (MoPrP^-/-^ D_162_GoPrP^+/-^). The absence of the murine PrP ORF in the transgenic mice was confirmed by PCR amplification using the primers 5’- TAGATGTCAAGGACCTTCAGCC-3’ and 5’-GTTCCACTGATTATGGGTACC-3’. Later, MoPrP^-/-^ D_162_GoPrP^+/-^ were crossed to produce homozygous MoPrP^-/-^ D_162_GoPrP^+/+^ mice for the transmission experiments.

### Analysis of PrP^C^ expression in OvPrP-Tg532 transgenic mice

Whole mice brains were homogenized in extraction buffer (0.5% NP-40, 1% sodium deoxycholate, 10 mM EDTA in phosphate-buffered saline [PBS], pH 7.4, with Complete protease inhibitor cocktail (Roche)). Samples were precleared by centrifugation at 2,000 X g for 5 min, added an equal volume of SDS reducing sample loading buffer and boiled for 5 min before loading onto an SDS-12% polyacrylamide gel. For immunoblotting experiments, the monoclonal antibody 12B2^25^ was used at a concentration of 1 µg/ml. This antibody recognizes the 93-WGQGG-97 epitope of the goat PrP sequence. Immunocomplexes were detected using horseradish peroxidase-conjugated anti-mouse IgG. Immunoblots were developed with enhanced chemiluminescence.

### Transmission studies

OvPrP-Tg532 and OvPrP-Tg501 (expressing wild type goat PrP^15^) transgenic mice were challenged with a collection of prion agents (see Table 1 for details). Inocula were prepared from infected brain tissues as 10% (wt/vol) homogenates in 5% glucose.

Groups of 6 to 9 individual identified animals (6 to 7 weeks old) were anesthetized and intracerebrally inoculated with 20 µl of 10% brain homogenate in the right parietal lobe, using a 25-gauge disposable hypodermic needle. As a control, 6 or 7 animals of each line were inoculated with healthy goat brain to discard the appearance of spontaneous prion disease. Mice were observed daily and their neurological status assessed. When the progression of prion disease was evident, or at the end of their life span (beyond 650 days of age), mice were euthanized for ethical reasons. During necropsy, part of the brain was harvested at 20 °C for determination of the presence of PrP^res^ by WB. Survival time was expressed as the mean number of survival days post-inoculation (dpi) for all the PrP^res^-positive mice. Attack rate was determined as the proportion of PrP^res^-positive mice among all the mice inoculated. The rest of the brain was fixed by immersion in 10% formalin to quantify spongiform degeneration by histopathology and PrP^res^ accumulation by immunohistochemistry (IHC).

### WB analysis

Brain tissue was homogenized in 5% glucose in distilled water in grinding tubes (Bio-Rad) and adjusted to 10% (w/v) by using a TeSeE™ Precess 48TM homogenizer (Bio-Rad) following the manufacturer’s instructions. To determine the presence of PrP^res^ in transgenic mouse brains, 100 μL of 10% brain homogenate were analyzed by WB as previously described^14^. For immunoblotting, membranes were incubated with Sha31 PrP monoclonal antibody (epitope 148-YEDRYYRE-155 of the goat PrP sequence)^26^ at a final concentration of 1 μg/mL. Immunocomplexes were detected with horseradish peroxidase- conjugated anti-mouse IgG (Amersham Pharmacia Biotech) and blots were developed with chemiluminescent substrate ECL Select (GE Healthcare Amersham Biosciences). Images were captured using ChemiDoc XRS + System and then processed using Image Lab 5.2.1 Software.

### Histological analysis

All procedures concerning the histopathological analysis of mouse brains were performed as previously described^27^. Mouse brain samples were fixed in neutral-buffered 10% formalin (4% 2-formaldehyde) during necropsy and paraffin embedded. 4 µm-thick tissue slices were stained with hematoxylin and eosin. Lesion profiles of the brains were established following published standard methods^28^. Semi-quantitative scoring of vacuolation from 0 to 3 in different brain areas was done to construct the vacuolation profile. The brain areas analyzed were the cerebral cortex (CCtx); corpus striatum (Stri); hippocampus (Hpp); thalamus (Thal); hypothalamus (Hpth); midbrain (Midb); cerebellar cortex (CbCtx); and pons/medulla oblongata (PoMe). For each brain area, a single value was calculated as the average vacuolation score. In addition, the total vacuolation score was determined by adding the average scores for the different brain areas. The ratios of the average score for each area to the total vacuolation score were expressed as percentages and plotted against the brain areas to produce lesion profiles. For IHC demonstration of PrP^Sc^ accumulation, tissue sections were subjected to antigen retrieval and quenching of hydrogen peroxide, as described previously^29^ and incubated with monoclonal PrP antibody 2A11 (epitope 163-QVYYRPVDQ-171 of the goat PrP sequence)^30^. The magnitude of PrP^Sc^ accumulation was scored from 0 (absent) to 3 (severe) in the abovementioned brain areas considering the following PrP^Sc^ types: intraneuronal (ITNR), intraglial (ITGL, combined intra-microglial and intraastrocytic), extracellular glia-associated (GLAS), fine particulate (PRTC), coalescing (COAL), linear (LINR) and plaque (PLAQ, vascular and non-vascular combined). For each PrP^Sc^ type, a single value was calculated as the average of the scores in the different brain areas, and the total brain PrP^Sc^ value was calculated as the sum of the different PrP^Sc^ averages, with a maximum potential score of 21. The average values for each PrP^Sc^ type were converted to percentages with respect to the total PrP^Sc^ content in each mouse, and these percentage values were used for graphical representation of PrP^Sc^.

## Supplementary Information

Supplementary Figure 1.

Supplementary Legend.

## Data Availability

No datasets were generated or analyzed during the current study.
