# Supplementary material for: Canine D163-PrP polymorphic variant does not provide complete protection against prion infection in small ruminant PrP context
Source: Sci Rep. 2021 Jul 12;11:14309. doi: 10.1038/s41598-021-93594-x (PMC8275588; doi:10.1038/s41598-021-93594-x)
Supplement: Supplementary file 2 — Supplementary Legend. [file 41598_2021_93594_MOESM2_ESM.docx]

Supplementary Figure 1. Full-length blots/gels corresponding to figures 1 and 2.
